# The Main Challenges in Systemic Lupus Erythematosus: Where Do We Stand?

**DOI:** 10.3390/jcm10020243

**Published:** 2021-01-11

**Authors:** Matteo Piga, Laurent Arnaud

**Affiliations:** 1 Rheumatology Unit, AOU University Clinic and University of Cagliari, 09042 Cagliari, Italy; matteopiga@unica.it; 2 Service de Rhumatologie, Hôpitaux Universitaires de Strasbourg, Université de Strasbourg, 67000 Strasbourg, France; 3 Centre National de Références des Maladies Systémiques et Auto-immunes Rares Est Sud-Ouest (RESO), 67000 Strasbourg, France

**Keywords:** systemic lupus erythematosus, review, disease activity, damage, glucocorticoids

## Abstract

Systemic lupus erythematosus (SLE) is an immune-mediated multi-systemic disease characterized by a wide variability of clinical manifestations and a course frequently subject to unpredictable flares. Despite significant advances in the understanding of the pathophysiology and optimization of medical care, patients with SLE still have significant mortality and carry a risk of progressive organ damage accrual and reduced health-related quality of life. New tools allow earlier classification of SLE, whereas tailored early intervention and treatment strategies targeted to clinical remission or low disease activity could offer the opportunity to reduce damage, thus improving long-term outcomes. Nevertheless, the early diagnosis of SLE is still an unmet need for many patients. Further disentangling the SLE susceptibility and complex pathogenesis will allow to identify more accurate biomarkers and implement new ways to measure disease activity. This could represent a major step forward to find new trials modalities for developing new drugs, optimizing the use of currently available therapeutics and minimizing glucocorticoids. Preventing and treating comorbidities in SLE, improving the management of hard-to-treat manifestations including management of SLE during pregnancy are among the remaining major unmet needs. This review provides insights and a research agenda for the main challenges in SLE.

## 1. Introduction

**Figure 1:**
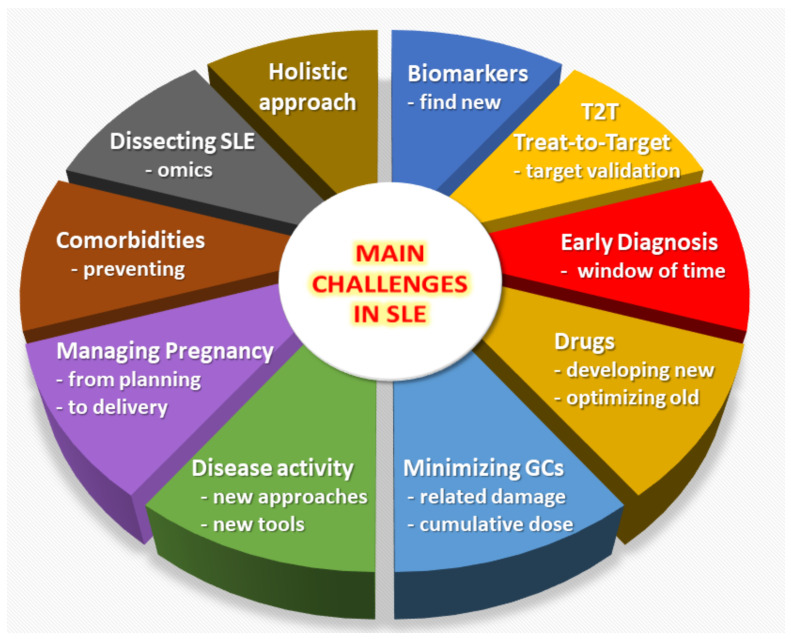
Overview of the main unmet needs in the field of Systemic Lupus Erythematosus (SLE).

Despite great improvements in treatment strategies leading to an improved prognosis [1,2,3], numerous challenges and unmet needs remain for the diagnosis and therapeutic management of Systemic Lupus Erythematosus (SLE) [4,5]. In this review we will provide an overview of the main unmet needs in the field of SLE (Figure 1), as a way to inform physicians, policy makers, funding institutions, and more generally the broad scientific community about the challenges and opportunities which remain in SLE research and clinical care.

## 2. Promoting Early Diagnosis

SLE is a complex disease with variable phenotypes and clinical manifestations. SLE onset is often insidious, with clinically evident disease developing over years. In addition, a variety of conditions may mimic SLE [6], including infectious and hematologic diseases, and for all these reasons the diagnosis may be delayed. It should not be surprising the median reported delay in SLE diagnosis is approximately 2 years.

It is common feeling that the early diagnosis of SLE can be beneficial by allowing early intervention and potentially improving short and long-term outcomes [5]. There is few evidence supporting this assumption and mainly derives from administrative database analysis showing that the patients with early diagnosis (<6 months between probable SLE onset and diagnosis) had lower rates of flares and hospitalizations compared with the late diagnosis patients (≥6 months) [7]. However, a clear identification of an early time frame between onset and diagnosis by which there are superior clinical responses and higher rate of remission in SLE patients has not been identified. Therefore, it is not proven that a window of opportunity really exists in SLE and a generally accepted definition of early disease is still lacking.

The identification of clinical and serological features useful in the differential diagnosis of patients with recent SLE onset [8] has facilitated the definition of classification criteria with greater sensitivity and specificity for early SLE compared to the previous validated criteria set [9]. Nonetheless, a recent single-center retrospective study suggested that 7–17% of patients diagnosed as having early SLE are not correctly classified using the EULAR/ACR 2019 [9], SLICC 2012 [10] and ACR 1982/1997 [11] criteria individually, while the combined use of all three sets of criteria ensured the classification of 94–98% of patients [12]. New tools for SLE classification are a major step forward for scientific purpose and may help in the earlier recognition of the disease, but they are not developed and should not be used for diagnostic purpose.

One major challenge is to implement effective strategies for earlier SLE diagnosis. These would take on greater value if a window of opportunity for SLE patients will be found and proven to improve outcomes including damage, death, recurrent flares, and Health-Related Quality of Life (HRQoL) measures.

## 3. Targeting Disease Remission (or Low Disease Activity)

Preventing flares and reducing damage accrual trough control of disease activity and reduction or withdrawn of glucocorticoids (GCs) are major challenges in SLE management and represents some of the objectives of the treat-to-target strategy for SLE (T2T/SLE) [13]. The T2T/SLE identified remission or low disease activity as the most important targets in SLE treatment, while it was recognized that there was no clear definitions for them. Recent advances in T2T/SLE include relevant definitions of clinical remission (CR) on treatment [14,15] and Lupus Low Disease Activity State (LLDAS) [16]. These definitions recognize the importance of durable absence or residual of disease activity measured using validated tools (SLEDAI, PGA), together with a stable treatment with antimalarials and/or immunosuppressants and a low GCs dose (prednisone ≤5 mg/day in CR and ≤7.5 mg/day in LLDAS). Although there is an ongoing debate around the potential overlap between CR and LLDAS definitions [17], they have been widely studied and resulted predictive of lower damage accrual in both newly diagnosed and long-standing SLE cohorts [18,19,20,21]. Interestingly, CR and LLDAS resulted independently associated with lower early damage accrual in an inception SLE cohort [22], confirming that CR is recommended as the primary treatment target in SLE and LLDAS represents a valid alternative also in the early stage of SLE management. Recently, the LLDAS has been prospectively validated as a SLE treatment endpoint in a multicenter international cohort demonstrating significant protection against flare and damage accrual [23].

Although LLDAS may represent a sufficiently validated outcome to be applied in clinical practice and trials, we still believe that treatment in SLE should aim at remission unless otherwise possible. Therefore, a major challenge is represented by the need to adequately validate existing definitions of CR in order to identify an attainable remission treatment endpoint, which should be indeed predictive of outcomes including damage, recurrent flares and death. Moreover, further data are needed on the role of CR and LLDAS in predicting better HRQoL outcome.

## 4. Considering New Ways to Assess Disease Activity

The quantification of disease activity in SLE represents a complex multi-dimensional concept, encompassing the physician evaluation of specific clinical manifestations attributed to SLE, the efficacy and response to prescribed medications and the patient personal feelings.

There are several physician-centered indices for disease activity assessment in SLE. Well-established measures exist to assess disease activity in specific organ (e.g., the Cutaneous Lupus Erythematosus Disease Area and Severity Index) but lack in others (e.g., musculoskeletal or renal manifestations). On the other hand, several tools have been developed to assess the overall disease activity. The most used include the SLE disease activity index (SLEDAI) and its evolutions, the British Isles Lupus Assessment Group (BILAG) and its revision, the European Consensus Lupus Assessment Measure (ECLAM), and the physician’s global assessment (PGA) by visual analogue scale. None of them have shown sufficient accuracy and sensitivity to change to be used alone as primary endpoints in RCTs. The PGA also suffers from reduced reliability suggesting the major need for standardization of its scoring [24,25]. We have therefore initiated an international collaboration to standardize the rating of the PGA in SLE (the PISCOS Study). Accordingly, novel composite outcomes such as the SLE responder index (SRI), which is based on the improvement of the SLEDAI with no worsening of the BILAG and the PGA, have appeared. Despite being considered more accurate in evaluate responsiveness to treatment, the SRI carries disadvantages of the individual indices from which it is composed, not least the need for clinician to judge if each manifestation is due to SLE or not. Recently, the SLE disease activity score (SLE-DAS), a continuous global score showing higher sensitivity to change and specificity than SLEDAI-2K [26], has been developed and is waiting for extensive validation. The patient component of disease assessment in SLE is not straightforward as patients tend to assess fatigue and pain, which are hardly related to disease activity.

Lupus patients and physicians are facing the need for more objective, reliable and reproducible ways to assess disease activity. Identifying new biomarkers of overall and organ specific disease activity and implementing their use in composite index may represent a major step forward. The application of deep machine-learning approaches would be helpful in the early identification of unfavorable individual patient trajectories among large SLE cohorts.

## 5. Minimizing the Use of Glucocorticoids

GCs still play a pivotal role in the treatment of SLE, especially in case of severe manifestations. However, several studies have emphasized the detrimental effects of chronic GCs therapy, particularly the increased risk for irreversible organ damage accrual. It has remained unclear which, if any, daily prednisone (equivalent) dose best prevent damage. Although <7.5 mg/day seem to minimize risk, even lower daily doses (4.4–6 mg/day) have been associated with a significant increase of damage [27]. In a recent multicenter Italian inception study, GC-related damage was independently associated with cumulative dose and steadily increased over time despite the reduced median daily prednisone dose below 5 mg since 12-month of follow-up [28]. However, it is not yet understood if and when GSs can be withdrawn [29]. In a survey by the SLICC group, almost 33% of patients never discontinued GCs after a mean follow-up of 7.26 years [30]. An observational study suggested that GC withdrawal is an achievable goal in SLE and may be attempted after a long-term remission or LLDAS to protect the patient from disease flares [31]. Contrarily, a randomized control trial (RCT) showed that patients with quiescent SLE who discontinued low-dose prednisone (5 mg/day) experienced significantly more flares than those who maintained this treatment [32].

Several challenges about the use of GCs in SLE emerged from these findings. Future RCTs should specifically address strategies to design effective GC tapering scheme enabling the use of the minimal possible dose of GCs for the shortest duration while minimizing the risk of flare. Moreover, when testing the efficacy of newly developed medication for the management of SLE, steroid sparing should be included in the assessment by means of cumulative GC doses or GC-related adverse events.

## 6. Developing More Effective Drugs and Optimizing the Use of Those Currently Available

The therapeutic management and global prognosis [33] of SLE have profoundly evolved over the years [2]. Following the discovery of GCs by Hench in the 40′, post-WW2 chemistry has brought many conventional immunosuppressive agents such as cyclosporine, azathioprine, cyclophosphamide, and more recently mycophenolate mofetil. Some adverse events have also taught us that some treatments can paradoxically induce lupus [34]. Antimalarials, the mainstay of SLE treatments have very favourable properties in lupus, but their efficacy to control disease activity and prevent flares is limited when used alone [35]. This has led to the need for the development of new treatments in SLE [36,37,38]. Unfortunately, effective therapeutics beyond GCs and classical immunosuppressive agents are limited [3]. Randomized controlled trials of rituximab and of at least 18 other molecules have failed in SLE, mostly due to issues associated with disease heterogeneity and trial design [39]. Therefore, there is only weak evidence upon which to base recommendations in many situations [40]. Optimizing the use of currently available therapeutics may represent a breakthrough. Belimumab has recently been tested in a 2-year RCT (BLISS-LN) in lupus nephritis and proved safe and effective when associated with the standard of care, while so far it was tested only in patients without active nephritis [41]. In an observational prospective study (BeRLiSS) treatment with belimumab early in the disease lead to better outcomes [42], which may suggest addressing the use of this agent as part of the first-line therapy for selected patients in innovative RCTs. Moreover, it appears urgent to develop more effective treatments in SLE, either through innovative trials of new agents [43] or of immunosuppressive drugs previously not tested in SLE (e.g., repository trials). Voclosporin, a next-generation calcineurin inhibitor, added to standard of care for induction therapy of active lupus nephritis resulted in a superior renal response but higher rates of adverse events including death were observed [44] Among the most recent advances is the better understanding of the role of interferons in the pathogenesis of SLE, which allowed for the development of drugs directly or indirectly targeting these pathways, such as interferon receptor blockers [36] or JAK inhibitors. Cellular therapy has shown interesting preliminary data and should also be improved [45] while new approaches, such as the use of low-dose IL-2 to expand regulatory T cells have emerged and appear promising [46]. Altogether, it is crucial to optimize the use of currently available therapeutics and develop new molecules assessing their efficacy through adequately designed trials using validated and robust outcomes.

## 7. Dissecting the Heterogeneity of the Disease

Environmental factors play a significant role in SLE development [47] but the interplay between genetic and environmental factors remains poorly understood at the patient level [3]. Also, epidemiology studies across different ethnic backgrounds are needed to understand better the polygenic basis and environmental influences upon disease risk, phenotypes and prognosis [3,8]. A large amount of evidence highlight that SLE has 3–4 times higher incidence, higher rate of lupus nephritis, worse severity in terms of damage accrual, HRQoL outcomes and three times greater mortality among African-Americans and other ethnic groups then in Caucasians. Although the LUMINA and Hopkins Lupus cohorts in the USA proved that socio-economic status play a major role in such ethnic disparities it is also conceivable that biologic differences might be responsible for distinct phenotypes. The SLE burden, mortality, outcomes, and quality of care and insights into health disparities and possible remedies across different ethnic backgrounds have been reviewed elsewhere [48,49,50].

Understanding the genetic component of SLE is complex because most patients have polygenic disease [51,52,53]. Genome-Wide Association Studies (GWAS) have allowed the description of more than 100 susceptibility Single Nucleotide Polymorphisms (SNPs) for SLE [52,53]. Most of those SNPs individually confer only a slight increase in the risk of SLE, making them of limited clinical utility for the diagnosis of the disease. Also, variants identified by GWAS explain only a fraction of overall heritability of SLE. Therefore, there is a missing heritability which could be explained notably by epigenetics, which remains poorly known in SLE. Finally, although very rare, the monogenic forms must be considered in the study of SLE genetics [6]. One major challenge is to develop efficient tools for characterizing patient and ethnic background heterogeneity using multi-omics. This will allow the development of personalized medicine for SLE patients. Currently, most teams are still using inaccurate biomarkers and the most recent advances are far from being implemented in most center.

## 8. Identifying Relevant Biomarkers for Individualized Treatment

**Figure 2:**
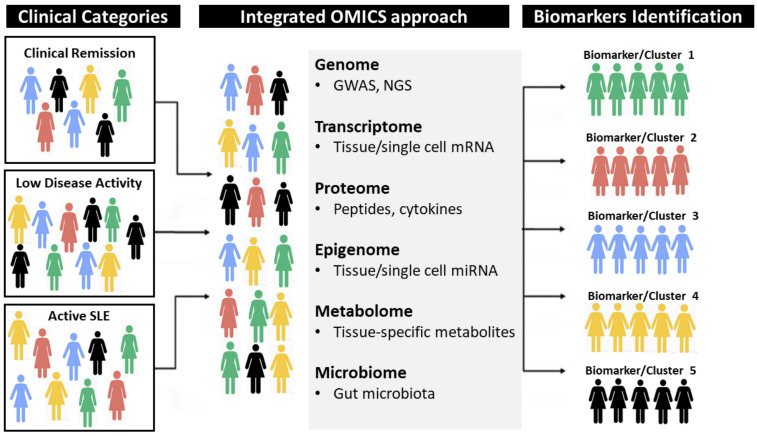
A hypothetical example illustrating how an integrated clinical and OMICS approach, driven by artificial intelligence, might help distinguishing homogeneous clusters from current heterogeneous phenotypes observed in Systemic Lupus Erythematosus (SLE). The example suggests that the way to identify new clusters with specific disease biomarkers should be tailored to the specific molecular events or pathways associated with disease activity and clinical phenotypes, providing a rationale for personalized therapeutic interventions.

Biomarkers to predict disease prognosis, disease remission and long-term adverse events are truly lacking in SLE [3]. The reliable identification of the right treatment for the right patient currently remains one of the most important challenges in SLE. In daily practice, the list of biomarkers which can be used in SLE has remained very limited, and includes mostly anti-dsDNA antibodies, complement factor proteins or leukopenia. Those are now insufficient to progress in the management of the disease and it is therefore crucial to identify reliable and advanced biomarkers. The era of multi-omics, biological analysis approach in which data from multiple “-omes” (such as the genome, transcriptome, proteome, epigenome, metabolome and microbiome), theoretically opens the door for highly integrated and individualized approaches [54]. At a proteomic level, cytokine profiles could be used as potential biomarkers. The most emblematic example is type I interferon gene signature found in the sera of 70–80% of active SLE patients. Blood interferon-alpha levels have been associated with the risk of subsequent flares in SLE [36]. Another approach is to assess urinary biomarkers in case of lupus nephritis [55], as this could be a better reflect of the local inflammation than when using blood-based markers. Pioneering studies tried to incorporate clinical characteristics into personalized immune-transcriptional data enabling patient stratification based on the immune networks best correlating with disease activity and providing a rationale for tailored therapeutic interventions [56]. One of the main current challenges is to integrate the vast amount of data available at the patient-level to make accurate predictions. This will require an in-depth interaction between clinical specialists, researchers in biomedicine and data scientists, with the help of artificial intelligence (Figure 2).

## 9. Managing Pregnancy in SLE

Pregnancy is a major challenge in SLE, and is generally managed by a tandem of a rheumatologist and obstetrician with significant experience with high-risk pregnancies, especially in case of antiphospholipid syndrome [57]. Pregnancy should be carefully anticipated in SLE, and pre-pregnancy multidisciplinary counseling is important to estimate the risk of maternal and fetal complications [58]. SLE is usually not associated with infertility unless the patient has been treated with cyclophosphamide, and ovarian protection strategies using GnRH agonists or ovarian preservation can be used, if needed. It is commonly recommended that the disease has been quiescent for at least 6 months (some experts suggest one year in case of lupus nephritis) before pregnancy is allowed because active SLE at the time of conception is a strong predictor of maternal and fetal complications. Also, positivity for antiphospholipid antibodies or SSA/SSB antibodies is associated with worst obstetrical outcomes, including prematurity, growth retardation, fetal death, neonatal lupus and congenital heat block [57,59]. One of the critical issues in managing women with SLE during pregnancy is choosing the right medication to treat the mother without harming the baby. Unfortunately, most medications used in SLE are potentially harmful or contraindicated during pregnancy and must be reviewed when planning pregnancy. However, there are safe options such as hydroxychloroquine (HCQ) and low dose aspirin (LDA) which demonstrated effective in reducing disease flares, obstetric and new-born complications [60,61]. Nevertheless, recent surveys found that the use of these drugs in pregnant women with SLE is still limited (HCQ 58% and LDA 25% of pregnancies) and should be increased [61,62]. Among the main challenges, ensuring fertility and proper course of pregnancy is of outstanding importance, especially in case of antiphospholipid syndrome, and remains one of the most important clinical challenges in SLE.

## 10. Preventing Comorbidities

Comorbidities, such as cardiovascular disease (CVD) and infections, are major responsible of increased mortality in patients with SLE. CVD is the leading cause of mortality in SLE regardless of time to occurrence after diagnosis [63]. The higher burden of CVD in SLE patients is mosly related to accelerated atherosclerosis, which leads to CV events at an earlier age compared to the general population. Accelerated atherosclerosis is driven by the interplay between inflammation, autoimmunity, medications and traditional risk factors. No surprisingly, the traditional CV risk factors are not sufficient to fully explain the increased number of CV events observed in SLE [64], which leads to an underestimation of the actual risk using existing tools validated in the general population. Recommendations for the management of CV risk factors in SLE patients exists [65], including the widespread use of hydroxychloroquine [66]. A major challenge we have to face is the need of validated tools for estimation of the CV risk in SLE, which represents the first step for conducting therapeutic trials to provide more evidence-based data on how to manage CV risk in SLE patients.

Infections are risk factor higher than disease activity for mortality in SLE patients [63,67]. GCs use, immunosuppressive therapy and lupus nephritis are the most important risk factor for infections in SLE patients. GCs related risk of infection increases by 12% for each mg/day of prednisone, thus is already high at 7.5 mg/day which is considered relatively safe for damage accrual [68]. In a recent meta-analysis, GCs were associated with an increased risk of COVID-19 in patients with autoimmune diseases including SLE [69]. A number of prophylactic measures against infections should be recommended in SLE. A recent audit of the British Society for Rheumatology estimated 34.3% of SLE patients need to adopt extreme social distancing measures (“shielding”) to minimize the risk of SARS-Cov2 infection [70]. Vaccination campaign should be implemented as vaccine administration rates remain low in SLE, in particular for vaccine against pneumococcus and influenza [71]. These are inactivated vaccines and therefore can be used at any time in SLE even though their immunogenicity may be substantially reduced if patient is taking immunosuppressant or high dose GCs. On the other hand, live attenuated vaccines are contraindicated in patients taking more than 10 mg/day of prednisone or immunosuppressant. The risk of SLE flare after vaccination is not confirmed, but vaccination should be avoided in patients with active disease. In order to reduce serious infections, besides the requirement to reduce chronic use of GCs there is an urgent need to strengthen the immunization coverage in patients with SLE. New vaccine strategies need to be evaluated and validated specifically in SLE also given the forthcoming availability of vaccination against Sars-Cov2.

## 11. Favoring a Global and Comprehensive Disease Management

An important challenge in SLE is to favor holistic medicine, which is the use of therapeutic strategies that attempt to treat the patient as a whole person. Feedback from SLE patients is essential. Patient-reported outcomes (PROs) capture patients’ perceptions of their health condition, HRQoL [72], well-being, and other aspects. Those encompass many crucial domains such as fatigue, anxiety, and depression among many others [73]. The use of PROs in daily clinical practice currently remains limited while those tools are essential for better characterizing the impact of SLE at the individual patient level [74]. Of note, the management of common, hard-to-treat manifestations, such as fatigue and depression should be clarified in SLE, according to EULAR [3]. For instance, we found that fatigue was reported by more than two-thirds patients and severe fatigue by more than one third in the large international FATILUP study [73]. We have also shown that the association between fatigue, anxiety and depression is very strong in SLE patients with inactive disease [75]. Therefore, we should conduct more and better designed trials to evaluate psycho-behavioral interventions as well as pharmacological interventions for the management of fatigue in SLE, targeting depression and anxiety. In SLE, just as in any other chronic disease, the proportion of patients not adhering fully to the prescribed treatments is very high [76] and can lead to apparently refractory disease [77]. The main predictors of non-adherence are a younger age, non-white ethnic background, low social-economic level, lower education level, unemployment, never-use of GCs, polymedication, mood disorders such as depression and rural residency [76,78]. Non-adherence contributes to worse patient outcomes, including an increased number of flares, visits to emergency departments and, importantly, mortality [76]. Also, disease prevalence, activity and severity is strongly increased in SLE smokers compared to non-smokers [79], while therapeutic responses are decreased [80]. It is therefore crucial to encourage SLE patients to stop tobacco. Also, physical inactivity is common in SLE with up to 72% of patients who do not meet the WHO recommendations [81]. Systematic reviews suggested that exercise reduces fatigue and depression, improves cardiorespiratory capacity without affecting disease activity [82,83]. Altogether, SLE should be managed globally as a chronic disease, understanding the patient’s perspective in her own holistic context.

## 12. Conclusions

Altogether, these challenges may be considered as an SLE roadmap for clinicians, researchers and health policy makers who wish to contribute to an improved and integrated management of this rare and complex disease.
